# The Structure and Mechanical Properties of FeAlCrNiV Eutectic Complex Concentrated Alloy

**DOI:** 10.3390/ma18153675

**Published:** 2025-08-05

**Authors:** Josef Pešička, Jozef Veselý, Robert Král, Stanislav Daniš, Peter Minárik, Eliška Jača, Jana Šmilauerová

**Affiliations:** 1 Department of Physics of Materials, Charles University, 12116 Prague, Czech Republic; jozef.vesely@matfyz.cuni.cz (J.V.); robert.kral@matfyz.cuni.cz (R.K.); peter.minarik@matfyz.cuni.cz (P.M.); eliska.jaca@matfyz.cuni.cz (E.J.); jana.smilauerova@matfyz.cuni.cz (J.Š.); 2 Department of Condensed Matter Physics, Charles University, 12116 Prague, Czech Republic; stanislav.danis@matfyz.cuni.cz

**Keywords:** eutectic complex concentrated alloys, microstructure, mechanical properties

## Abstract

In this work, the microstructure and mechanical properties of the FeAlCrNiV complex concentrated alloy (CCA) were studied in the as-cast and annealed states. The material was annealed at 800 °C for 16 days to test microstructure stability and phase evolution. It was found that the microstructure does not differ in the two investigated states, and the results of differential scanning calorimetry and dilatometry showed that there is almost no difference in the thermal response between the as-cast and annealed states. Both investigated states exhibit eutectic structure with bcc solid solution and ordered phase with B2 symmetry. In a single grain, several regions with B2 laths in the bcc matrix were observed. Inside the B2 laths and in the bcc matrix, bcc spheres and B2 spheres were observed, respectively. All three features—laths, matrix and spheres—are fully crystallographically coherent. Nevertheless, in the adjacent region in the grain, the crystal structure of the matrix, laths and sphere changed to the other structure, i.e., the characteristics of the microstructure feature with B2 symmetry changed to bcc, and vice versa. Compression deformation tests were performed for various temperatures from room temperature to 800 °C. The results showed that the material exhibits exceptional yield stress values, especially at high temperatures (820 MPa/800 °C), and excellent plasticity (25%).

## 1. Introduction

A promising group of structural materials called complex concentrated alloys (CCAs) are attracting the attention of the metallic materials community. CCAs comprise several elements (usually 3 or more), typically in near-equiatomic ratios. Therefore, no constituting element can be considered the principal one, in contrast to classical alloys [1]. CCAs expand the concept of so-called high-entropy alloys (HEAs), which are multicomponent alloys of five or more elements, with near equiatomic ratios and one single solid solution, usually bcc or fcc symmetry. Moreover, the term CCAs is not limited to alloys with a single-phase solid solution structure, as was the case in the early studies of HEAs [2]—CCAs can comprise two or more phases. The systematic research of these materials began in the early 1990s (several dozen papers per year). Research has intensified in recent years (more than a thousand papers per year). These materials are generally investigated for their high strength and hardness at high temperatures [3,4,5,6,7,8,9,10,11,12,13,14]. On the other hand, most multicomponent alloys suffer from limited plasticity, especially at room temperature [15,16]. To ensure both strength and plasticity, Lu et al. [17] designed a novel strategy to tackle this problem. They proposed CCAs with a eutectic structure, in which two phases alternate: one with a solid-solution structure that promotes ductility and the other with an ordered (intermetallic) structure that promotes strength even at elevated temperatures. In recent years, many eutectic CCAs have been investigated [18,19,20,21,22,23,24,25,26,27,28,29,30,31], for example, Al14Co28Cr28Ni30 consisting of fcc and ordered bcc B2 phases [18], CoCrFeNiNbx with fcc and Laves phases [19], CoCrFeNiNbx with soft fcc and hard Laves phase [20]. The findings of these papers present a very good combination of high strength and sufficient plasticity of the studied materials. This approach is inspired by the design of Ni-based superalloys which contain a combination of a fcc phase and an ordered L12 structure. As the intermetallic phase is in the form of cuboids with coherent interfaces with the fcc matrix, these alloys possess excellent mechanical properties even at elevated temperatures [32].

The research of our group in the past was focused on the study of iron aluminides. Therefore, our previous materials designs were based on iron aluminides [28,29,30,31,33]. We intended to introduce ordered phases like B2 (FeAl) or D03 (Fe3Al) into the structure of the studied materials. The structure of equiatomic FeAlCrV and FeAlCrMo alloys consists of bcc solid solution and ordered D03 phase with a minority of particles [28,29]. These materials exhibit excellent strength but insufficient plasticity. Another direction of our research focused on the study of FeAlCr_x_Ni_x_V_x_ and Fe3AlN_x_iCr_x_V_x_ materials, where 5 at. % ≤ x ≤ 20 at. % [30,31,33]. Kratochvíl et al. [33] have shown that it is possible to use the Labusch solid solution strengthening model [34] for HEAs in which one element has a concentration lower than 5 at. %. In this case, the element with a concentration below 5 at. % is considered as the solute, while the remaining elements are considered as the solvent (matrix).

The main goal of the work is to study the structure and stability depending on the temperature of the equiatomic FeAlCrNiV alloy. The mechanical properties of the alloy were also tested. The design of the composition of this alloy is stimulated by the fact that there is a pair of elements, Ni and Al, with a strong affinity to form an ordered phase. The formation of an ordered NiAl phase causes depletion of Ni and Al, and the composition in the vicinity of the ordered phase will be favorable for the formation of the bcc phase. The consumption of Ni and Al during the formation of the ordered phase is high, and therefore, we expect that the alteration of the phases will be achieved at a nanoscale measure. The main idea is to achieve a microstructure combining a disordered bcc and the ordered NiAl phase, as it should provide both ductility (due to the disordered bcc phase) and strength (due to the NiAl phase). Moreover, the NiAl phase has very good thermal stability [35], which is also crucial. There are two aims of this study. The first is to obtain a eutectic structure of the material, which is stable with temperature. The second aim is to reach sufficiently high strain to fracture at RT. Both of these conditions are very important from the point of view of application use.

## 2. Experimental

The melting process was carried out under intensive stirring during melting in a medium-frequency vacuum induction furnace with a maximum melting weight of about 900 g. The medium-frequency vacuum furnace was equipped with a specialized feeding system for the gradual addition of elements. The purity of all the elements used was 99.8 at. % or better. In the case of Fe and V, 0.1 at. % of C was detected. The constituent elements were added to the melt according to their oxygen affinity, and the least reactant was melted first. The vacuum pressure in the furnace chamber was 4 Pa for each melt. When the batch was completely melted, the furnace was filled with argon gas to 60,000 Pa and the alloy was cast into a mold in this protective atmosphere. The cast molds were left in a closed furnace to minimize the cooling rate of the ingots.

Phase transformations were investigated by differential scanning calorimetry (DSC) and dilatometry. Microstructure analyses were performed using X-ray diffraction (XRD), scanning and transmission electron microscopy (SEM, TEM), and energy dispersive X-ray spectroscopy (EDX).

DSC measurements were performed using a Netzsch DSC 404 C Pegasus (Selb, Germany). Samples for DSC were cut and polished to a flat shape with a weight of approximately 40 mg. Linear heating from room temperature to 1000 °C with a heating rate of 10 °C/min was used in a pure Ar atmosphere. The heat flow was calibrated using a sapphire standard; the procedure is described, e.g., in [36].

Dilatometry was measured on a Linseis L75 PT vertical dilatometer with cuboidal samples with planparallel bases. Dilatometry measurements were carried out from room temperature to 1000 °C. The conditions of the experiment were identical to those of DSC, i.e., the linear heating rate was 10 °C/min and a protective Ar atmosphere was used. For both experimental methods (DSC and dilatometry), two identical heating runs were performed to assess the reversibility of the transformations.

Phase analysis and lattice constant measurements on powdered samples (powder was prepared by drilling the material using a diamond drill bit) were performed using an X-ray diffractometer (Bruker D8 Advance, Ettlingen, Germany) with CuKα radiation. The diffracted radiation was collected by an energy-dispersive detector (SolX). The profile analysis of the diffraction patterns was performed using the Rietveld method (FullProf). The cross section of the primary X-ray beam on the sample surface was held constant by automatic divergence slits. The sample was rotated during the measurement (30 rpm). The time step for one scan was set to 5 s. However, several scans were averaged with the total time per step of 60 s.

The samples for SEM were cut from the ingot, embedded in a conductive epoxy resin, and mechanically polished using SiC papers, with the grit size gradually decreasing down to 50 nm. SEM investigation was performed on a ZEISS Auriga Compact (Oberkochen, Germany) equipped with an EDAX EDX spectrometer. TEM observations were carried out on a JEOL JEM-2200FS (Peabody, MA, USA) operating at 200 kV and equipped with an EDAX EDX detector. Disks about 0.5 mm in thickness and 3 mm in diameter were cut from the material and subsequently polished on SiC papers to a thickness of about 100 µm. Finally, Struers Tenupol 5 was used to electropolish the specimens at 15 V and −30 °C in the 17% solution of HClO4 in methanol to prepare TEM foils. Electropolishing in 20% solution of HNO3 in methanol, a usual electrolyte for FeAl alloys, resulted in preferential etching of the bcc phase.

For mechanical compression tests, 5 mm × 5 mm × 7.5 mm square prisms were cut from the material using a diamond wafering blade. These samples were deformed using a digitally controlled testing machine (INSTRON 1186R, Norwood, MA, USA) in uniaxial compression at room temperature, 200 °C, 400 °C, 600 °C and 800 °C. The strain rate was 1.2 × 10^−4^ s^−1^.

Structural and mechanical properties were studied in the as-cast state and after annealing at 800 °C for 16 days. The temperature value was chosen in correspondence with the highest temperature of the mechanical tests. The aim of annealing was to homogenize the material and to prove the stability of the structure. The samples were aged at this temperature in evacuated (10^−6^ mbar) quartz ampoule. After 16 days, the furnace was turned off and after reaching RT of the samples, and the aging was finished.

## 3. Results and Discussion

### 3.1. Material

**Figure 1:**
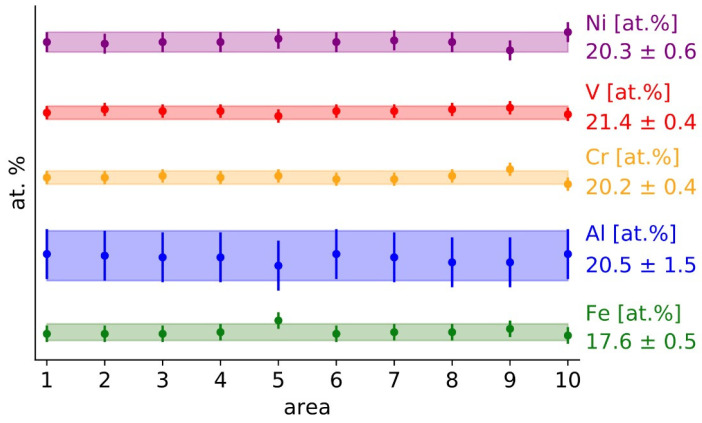
Composition of the FeAlCrNiV alloy in the as-cast state. Markers denote the amount of the element in the selected area, whereas the shaded region represents the average value and its deviation.

The composition of the cast material was determined using EDX spectroscopy in SEM. Three specimens were prepared from various randomly selected positions in the ingot and at least three areas of 500 × 500 µm^2^ were analyzed from each specimen. The resulting values of 10 analyses are shown in Figure 1.

The material shows excellent homogeneity in concentration and the resulting composition is consistent with the nominal composition.

### 3.2. Microstructure

**Figure 2:**
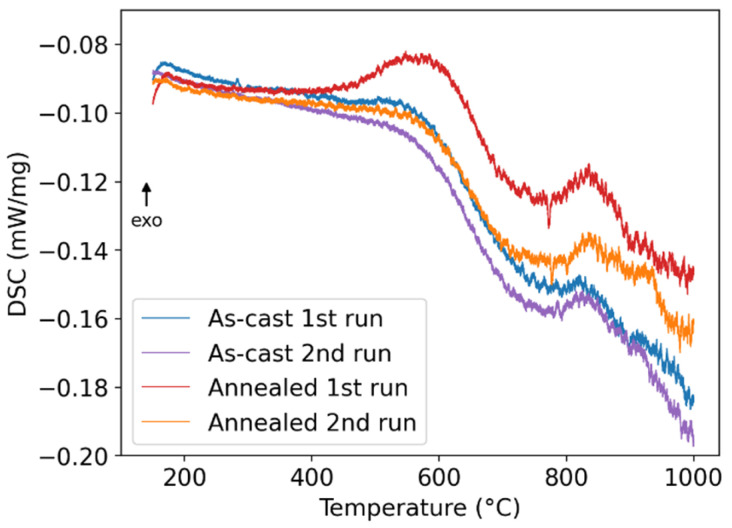
The DSC curves of the FeAlCrNiV alloy in the as-cast and annealed (800 °C/16 days) conditions.

**Figure 3:**
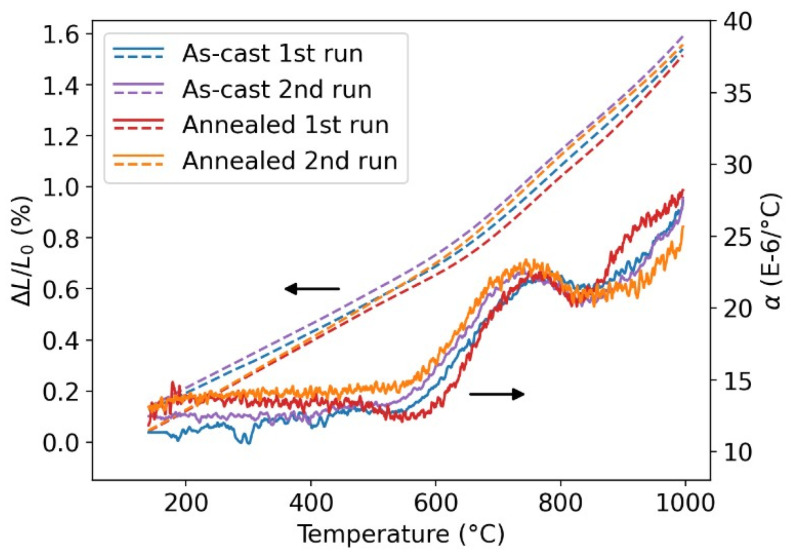
Results of dilatometry of the FeAlCrNiV alloy in the as-cast and annealed (800 °C/16 days) conditions.

The DSC and dilatometry results for the FeAlCrNiV alloy are presented in Figure 2 and Figure 3, respectively. Two heating runs for each measured sample are shown, which is useful for determining whether a transformation is reversible.

The DSC curves for the first and second heating runs of the as-cast sample (blue and violet curves in Figure 2, respectively) are nearly identical, indicating that the predominant thermal processes occurring during linear heating are largely reversible. The only notable difference is a subtle exothermic bump in the first heating run (blue curve) between approximately 500 °C and 600 °C. Beyond this range, both curves exhibit a deviation in the endothermic direction, with an onset around 550 °C. The shape of the endothermic signal from 550 °C to the maximum measured temperature of 1000 °C suggests the presence of two overlapping thermal events. The broad nature of these peaks implies that both processes occur gradually over a wide temperature range.

After annealing at 800 °C for 16 days, the thermal behavior of the alloy changes slightly during the first heating run (red curve in Figure 2). A more pronounced exothermic event is observed, starting at approximately 450 °C. This process is likely the same as the one responsible for the exothermic bump in the DSC curve for the as-cast condition (blue curve in Figure 2), though more intense in the annealed sample. The second heating run of the annealed condition (orange curve in Figure 2) is very similar to the second run of the as-cast sample, indicating structure stability even after a long-term annealing at an elevated temperature.

The broad endothermic processes can be attributed to the combined effects of σ-phase dissolution and the decomposition of the bcc matrix into two distinct bcc phases with very close lattice parameters. Evidence for these two transformations is provided by XRD analysis comparing the as-cast and annealed (800 °C) conditions. The exothermic bump is consistent with the precipitation of the σ-phase, which is expected to occur within the temperature range of 450–600 °C [37,38]. The enhanced intensity of the exothermic peak in the annealed conditions can be explained by the dissolution of the σ-phase during annealing at 800 °C, leading to greater reprecipitation upon reheating. The absence of this exothermic event during the second heating runs is likely due to reprecipitation of the σ-phase during the slow cooling that follows the first DSC cycle.

Figure 3 shows the thermal expansion of the FeAlCrNiV alloy in both as-cast and annealed states. Dashed lines correspond to the relative length change *L*/*L*_0_, while solid lines represent the coefficient of thermal expansion α. The coefficient of thermal expansion α is defined as follows:

(1) $$\alpha \;=\;\frac{1}{L_{0}}\;\frac{\partial L}{\partial T}$$

where *L* is the length of the sample at temperature *T* and *L*_0_ is the initial sample length at room temperature.

In the as-cast condition, both heating runs are nearly identical (blue and violet curves in Figure 3). The material exhibits linear expansion up to approximately 550 °C, beyond which the expansion rate increases noticeably. This change is most clearly visible in the derivative curve (the coefficient of thermal expansion α, full lines in Figure 3). Two distinct increases can be observed: the first beginning at around 550 °C, and the second near 800 °C. These two increases in the thermal expansion rate suggest the presence of two thermally activated processes, consistent with the endothermic events observed in the DSC results.

The annealed condition (800 °C/16 days) shows a slightly different thermal response in the first heating run, which matches the DSC observations. First, a slight reduction in the rate of thermal expansion is observed, starting at approximately 450 °C, as shown by the full red line in Figure 3. Second, the increase in thermal expansion above 800 °C is steeper, which is again best visible on the coefficient of thermal expansion α. The second heating run of the annealed sample (orange curve) shows the behavior observed in both heating runs of the as-cast condition, confirming the reversibility of the involved processes.

**Figure 4:**
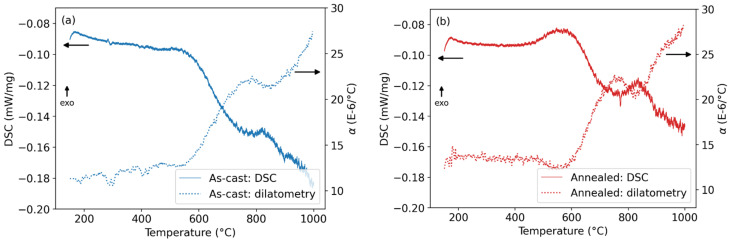
Comparison of DSC and dilatometry (coefficient of thermal expansion α) for the first runs of (**a**) the as-cast and (**b**) the aged condition of the FeAlCrNiV alloy.

Figure 4a,b compare the DSC signal with the coefficient of thermal expansion α for the first heating runs of the as-cast and annealed conditions, respectively. The correspondence between the two data sets is remarkably consistent. In particular, the exothermic event between approximately 450 °C and 600 °C (attributed to precipitation of the σ-phase) is accompanied by a reduction in the thermal expansion rate, which is especially evident in the annealed condition (Figure 4b). This is attributed to the lower molar volume of the forming σ-phase compared to the matrix phases (bcc and B2), which leads to a temporary suppression of overall dimensional growth. Furthermore, the endothermic processes occurring above 600 °C, namely the dissolution of the σ-phase and the decomposition of the bcc lattice into two distinct bcc phases with similar lattice parameters, result in an accelerated thermal expansion. Therefore, these transformations are associated with an increase in the sample volume, as reflected in the step-like rises in the thermal coefficients α.

To enhance the clarity of our interpretation, we now provide a more direct correlation between thermal events observed in DSC/dilatometry and the structural transformations evidenced by XRD.

**Figure 5:**
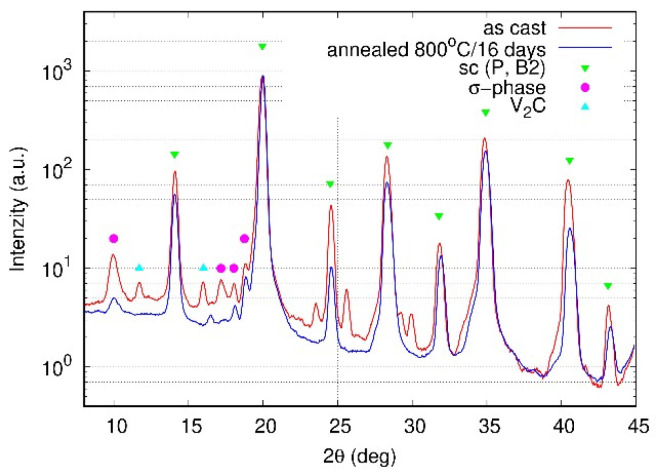
X-ray diffraction patterns of the as-cast and annealed sample measured using MoKα radiation.

The exothermic bump observed in the DSC curves between 450 °C and 600 °C, more pronounced in the annealed condition (Figure 2), is attributed to the precipitation of the σ-phase. In the same temperature range, a decrease in the coefficient of thermal expansion α is observed (Figure 3), consistent with the lower molar volume of the σ-phase compared to the bcc/B2 matrix. This identification of the phase transformation observed in DSC and dilatometry is supported by the presence of weak σ-phase peaks in the XRD patterns of the annealed sample (Figure 5) and by their absence in the as-cast sample.

**Figure 6:**
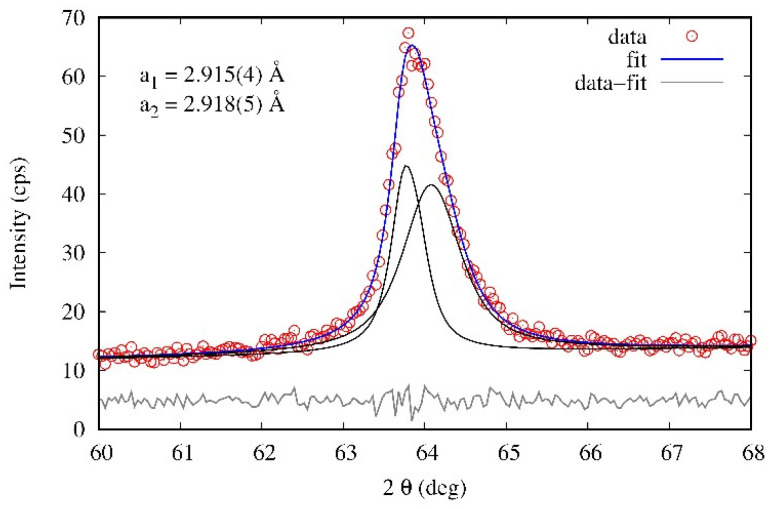
Decomposed diffraction profile of (200) reflection, annealed sample. Note the contribution of two slightly different cubic phases. Measured using CuKα radiation.

The broad endothermic signal above 600 °C in the DSC is associated with two overlapping events. The first one is the dissolution of the σ-phase, as evidenced by the disappearance of its characteristic peaks in the XRD pattern after annealing (Figure 5). The second one is the decomposition of the original bcc phase into two bcc variants, indicated by the XRD splitting of the (200) reflection into two peaks with slightly different lattice parameters (Figure 6). This decomposition is further supported by dilatometry, where a sharp increase in α occurs around the same temperature range, corresponding to an increase in sample volume due to lattice rearrangement.

The described sequence of transformations detected by DSC and dilatometry is thermodynamically consistent and validated by the reversible nature of the thermal responses in second heating runs and by XRD-detectable phase changes.

The DSC and dilatometry results demonstrate that the main transformations in the FeAlCrNiV alloy are largely reversible, as evidenced by the close agreement between the first and second heating runs in both measurement techniques. In addition to the exothermic event associated with σ-phase precipitation, presented only in the first heating, the thermal responses of the as-cast and annealed conditions are remarkably similar. Therefore, we may assume that the microstructure and phase composition of the FeAlCrNiV alloy is stable, since the thermal response of the material was not significantly influenced by the long-term annealing at an elevated temperature.

The X-ray diffraction patterns of the FeAlCrNiV alloy in the as-cast and annealed states are shown in Figure 5. The positions of all HEA peaks correspond to the cubic symmetry with primitive (sc—simple cubic) cubic lattice, which can be assigned to the ordered B2 structure as well. In addition to these cubic peaks, several small peaks around the maximal peak correspond to the σ-phase and V2C particles. σ-phase with similar lattice parameters is found in the binary systems Fe-Cr (a = 0.87995 nm, c = 0.45442), Fe-V (a = 0.8956 nm, c = 0.4627 nm) and Ni-V (a = 0.898 nm, c = 0.464 nm) [31]. The lattice parameter of the as-cast alloy is *a* = 0.2904(4) nm. After 800 °C/16 days aging, the initial cubic phase was decomposed into two cubic phases with nearly the same lattice parameter, as shown in Figure 6: a = 0.2915(4) and 0.2918(5) nm (black lines in Fig. 6 correspond to two cubic phases). 

**Figure 7:**
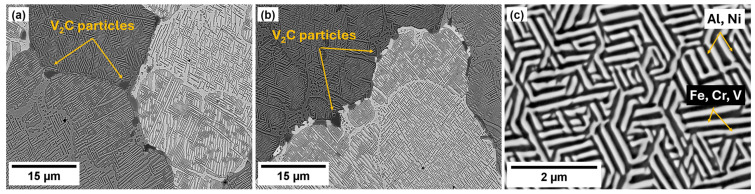
SEM micrographs of (**a**) the as-cast state, and (**b**), (**c**) the annealed state. Backscattered electron contrast.

Figure 7 shows the SEM micrographs of the as-cast and annealed states. The eutectic structure dominates the microstructure of both states, and there is nearly no difference in the morphology of phases, size of grains, and other structure characteristics. The grain size is in the range 100 µm–200 µm (Figure 7a,b). The eutectic structure comprises two alternating phases forming laths approx. 200–300 nm wide and 1000 nm long (Figure 7c). Note that this eutectic morphology does not change with annealing. Due to the different compositions of the two phases, different contrasts are observed in the backscattered electron image in SEM. The white and dark regions correspond to phases containing heavier and lighter elements, respectively. Figure 7a,b show that the grain boundaries are populated with particles, especially in the as-cast state. In the as-cast state, the size of these particles is approximately 1 µm and the distance between particles is approximately 10 µm (Figure 7a). After annealing, the number of particles decreased, but some of them became larger (Figure 7b). The occurrence of the V2C particle at the grain boundaries is very irregular. There are also very long parts of the boundaries without particles. Nevertheless, the volume fraction of particles in the as-cast and annealed states is comparable and is about 2%. Note that the phase analysis of both eutectic phases and particles is shown in the following TEM investigation.

**Figure 8:**
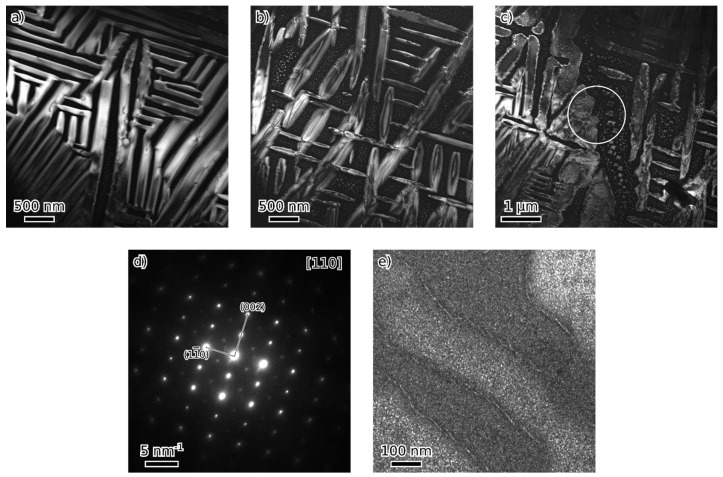
TEM micrographs of as-cast alloy—dark field (DF) images from (100) B2 spot showing two types of microstructures (**a**,**b**) and their transition (**c**). [110] The SAED pattern (**d**)—the circle in (**c**) marks the approximate size of SA aperture. DF image from (111) D03 spot (**e**).

**Figure 9:**
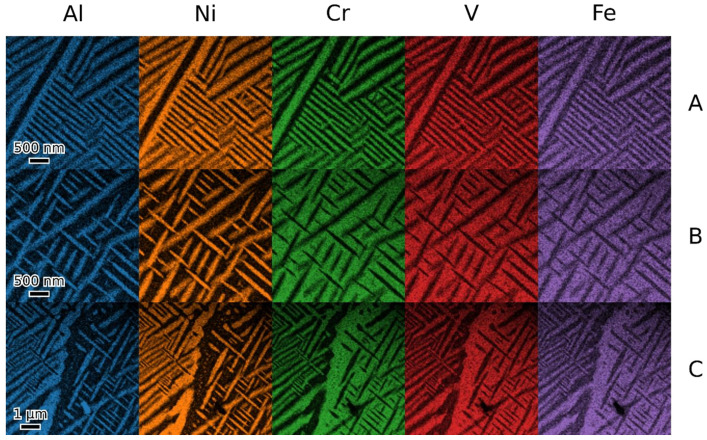
EDX elemental maps of the two types of microstructures of the as-cast state (**A**,**B**) and their transition (**C**).

**Figure 10:**
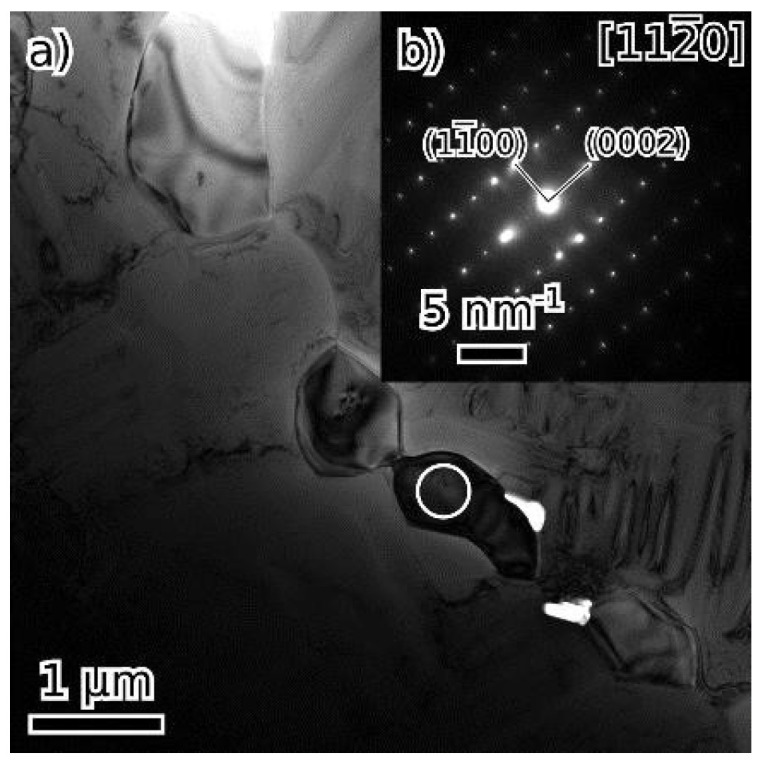
Particles of the V2C phase in the as-cast state (**a**), (**b**) shows the [11$\bar{2}0$] diffraction pattern from the particle marked by a circle.

**Figure 11:**
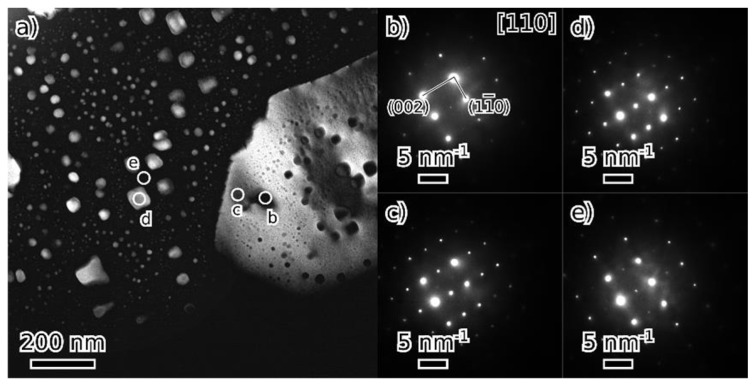
Dark field (DF) image (from (100) B2 spot) of a detail of a microstructure of the as-cast state (**a**); nanobeam diffraction (NBD) patterns in the [110] orientation (**b**–**e**) are from locations marked by circles.

**Figure 12:**
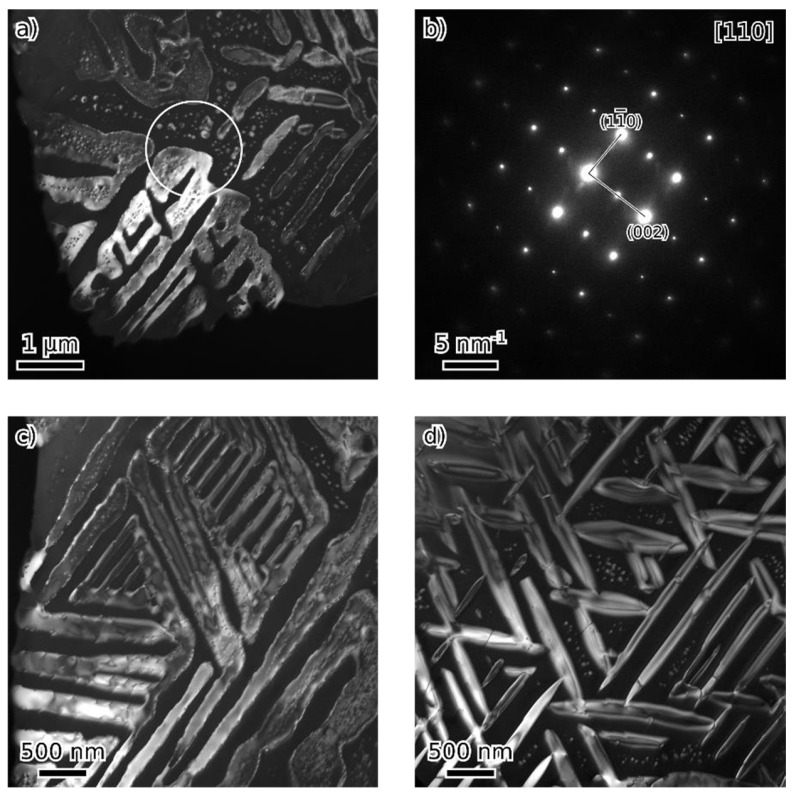
TEM micrographs of the annealed alloy—dark field (DF) images of the (100) B2 spot showing two types of microstructures (**c**,**d**) and their transition (**a**). [110] The SAED pattern (**b**)—the circle in (**a**) marks the approximate size of SA aperture.

**Figure 13:**
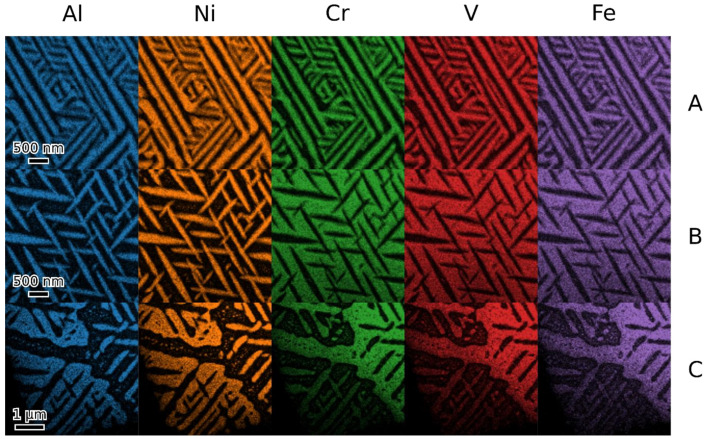
EDX maps of two types of microstructures of the annealed alloy (**A**,**B**) and the transition between them (**C**).

**Figure 14:**
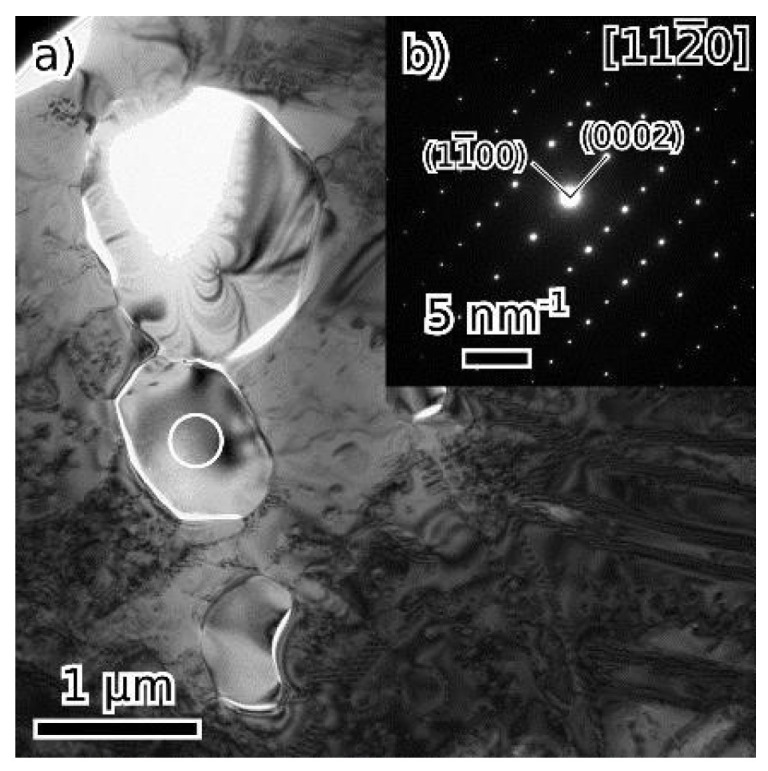
Particles of the V2C phase in the annealed alloy (**a**); (**b**) the [11$\bar{2}0$] diffraction pattern from the particle marked by a circle.

**Figure 15:**
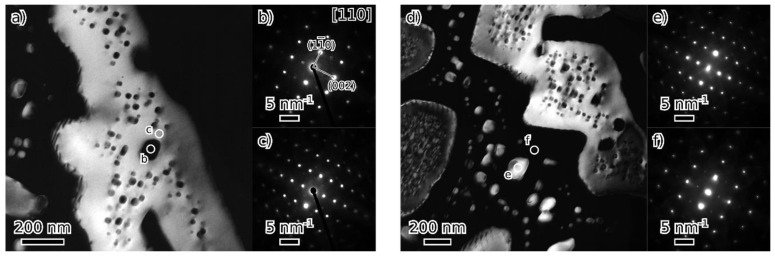
Dark field (DF) image (from (100) B2 spot) of details of a microstructure of annealed state (**a**,**d**); nanobeam diffraction (NBD) patterns in [110] orientation (**b**,**c**,**e**,**f**) are from locations marked by circles.

TEM observation of materials in the as-cast and annealed states showed a very similar structure of both states. Figure 8, Figure 9, Figure 10 and Figure 11 show the structure of the as-cast state, while Figure 12, Figure 13, Figure 14 and Figure 15 show the structure of the annealed state. Selected area electron diffraction (SAED) (Figure 8d and Figure 12b), dark-field (DF) imaging (Figure 8, Figure 11, Figure 12 and Figure 15) and nanobeam diffraction (NBD) (Figure 11 and Figure 15) revealed that two alternating phases have bcc and B2 structure. Energy dispersive X-ray (EDX) spectroscopy showed (Figure 9 and Figure 13 and Table 1) that the bcc phase is composed predominantly of V, Cr, and Fe, while the B2 phase is formed by Ni and Al. The NiAl B2 phase appears dark in SEM micrographs (Figure 7c) due to the low atomic number of Al. However, it is bright in (100) DF TEM images, since the (100) spot is forbidden in bcc but allowed in the B2 structure. Both phases are crystalographically coherent. More detailed investigation showed two kinds of areas: in some places (Figure 8a and Figure 12c), the B2 phase appears to be continuous (matrix), containing bcc as laths; in other places (Figure 8b and Figure 12d), the roles are reversed—bcc matrix, B2 laths. These areas are 20–100 µm in size, and several of them form a single crystallographic grain. The laths lay parallel to the (100) planes.

At higher magnification, both phases (matrix and laths) contain small (20–50 nm) spherical particles (Figure 11 and Figure 15) of the complementary phase (bcc in B2, B2 in bcc). These seem to have formed only at distances greater than about 100 nm from the phase boundary; therefore, thinner laths or parts of the matrix are without these particles.

The only difference between the as-cast and annealed alloy appears to be the presence of weak (111) D03 spot in the SAED pattern (Figure 8d) of the as-cast alloy. The corresponding DF image (Figure 8e) shows a nanoscale dispersion of D03 in both bcc and B2. This is not observed in the annealed alloy.

Particles at grain boundaries in both the as-cast and annealed alloy (Figure 10 and Figure 14, respectively) were identified by SAED as V2C (hexagonal lattice, a = 0.29 nm, c = 4.58 nm) [39].

The XRD analysis of the annealed state does not show the peaks corresponding to the grain boundary particles, but particle peaks are detected in the as-cast state, as shown in Figure 5. The reason for this is the random crystallographic orientation of the particles with respect to the direction of X-rays. In the case of the annealed state, many particles are not suitably oriented, so the volume fraction of reflecting particles is low. In the case of the as-cast state, several large particles with suitable orientation are sufficient to observe peaks from the particles.

The microstructures of both states have a nanoscale character. This fact could be related to the strong affinity of Ni and Al to form an ordered phase with B2 symmetry. On the other hand, after the depletion of Ni and Al, the prevailing concentrations of Fe, Cr, and V atoms form a bcc solid solution. Because the concentrations of elements in the corresponding phases (Ni, Al in B2 and Fe, Al, V in bcc) are very high (see Table 1), the consumption of these elements is also very high. That means that after the formation of one phase, the surrounding space is very quickly depleted of the elements needed for the growth of this phase, and the composition of the surrounding region is suitable for the formation of the second phase. As a result of this fact, the individual phases change in nanoscale measures.

**Figure 16:**
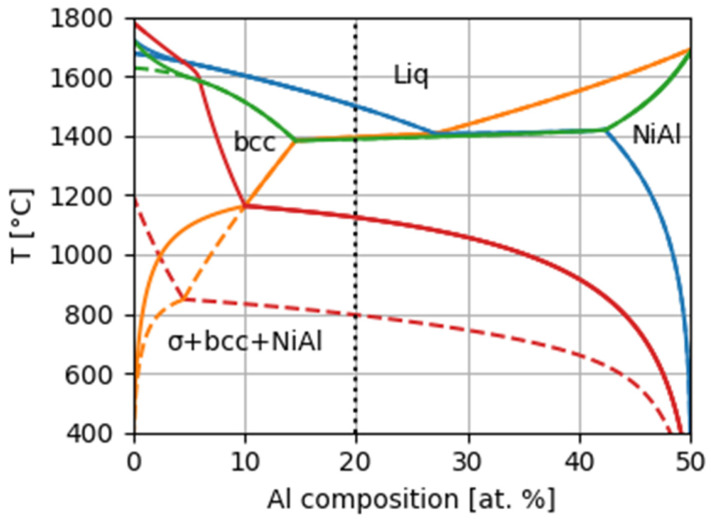
Phase diagram cut between the bcc and NiAl phases. Green, orange, blue and red lines mark zero-phase fraction lines for liquid, NiAl, bcc, and σ-phase, respectively. Dashed lines are calculated with σ-phase suppressed by 2.16 kJ/mol (see text).

Phase equilibria were calculated using Thermocalc software (version 2022a) and the TCHEA5 database [40,41]. As we previously reported [31], the stability of σ-phase in this composition range is overestimated by the TCHEA5 database. Table 1 reports the equilibria of the bcc and B2 phases, calculated with σ-phase rejected, along with the experimental values. It can be clearly seen that the experimental results are in good agreement with the Thermocalc predictions. Moreover, an isopleth (Figure 16) between the bcc and NiAl phases was calculated. The conditions for the concentrations of constituents (in atomic percents) in the alloy were as follows: x_Ni_ = x_Al_, x_Fe_ = x_V_ = x_Cr_. These conditions are consistent with the results of the chemical composition determined from EDX measurements (Table 1).

The X-ray results show the dissolution of the σ-phase after annealing at 800 °C for 16 days (Figure 5). This is further corroborated by the peak at 780–830 °C on the DSC signal (Figure 2). To accommodate these experimental results, the calculation was repeated with the Gibbs energy of σ-phase increased by 2.16 kJ/mol (65 kJ per mole of formula unit using the PHASE_ADDITION command). The results are shown as dashed lines in Figure 16.

The pseudobinary phase diagram in Figure 16 confirms our statement that the decomposition of the studied alloy has eutectic character.

### 3.3. Mechanical Properties

**Figure 17:**
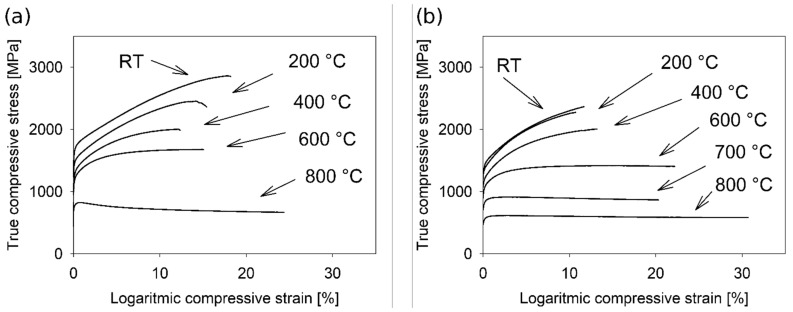
Deformation curves from compression tests for (**a**) the as-cast state and (**b**) the annealed state.

Figure 17 presents compressive true stress–logarithmic strain curves of the as-cast and annealed material at various temperatures of deformation (RT, 200 °C, 400 °C, 600 °C and 800 °C, in case of annealed material also 700 °C). The characteristic parameters of the mechanical properties, such as the yield stress (σ02), strain to fracture (εf), and the maximum stress (σM), for all temperatures of deformation are listed in Table 2. The yield stress values for all temperatures of deformation are very high compared with those of conventional alloys, especially at higher temperatures. Also, the strain to fracture values are sufficiently high for a good formability of the material (Table 2). In general, the high strength of the entropy alloys is very often correlated with the bcc structure, but in this case the strain to fracture has lower values, especially at RT. In our case, the strain to fracture reaches very good values compared to other HEAs [3,4,5,6,7,8,9,10,11,12,13,14] (Table 2 and Table 3), especially at low temperatures (more than 10%). The eutectic characteristic of the structure of the studied material comprises many features in the nanoscale range. This fact results in more homogeneous inner stress, leading to a lower probability of crack formation. Consequently, very good strain to fracture values can be achieved.

**Figure 18:**
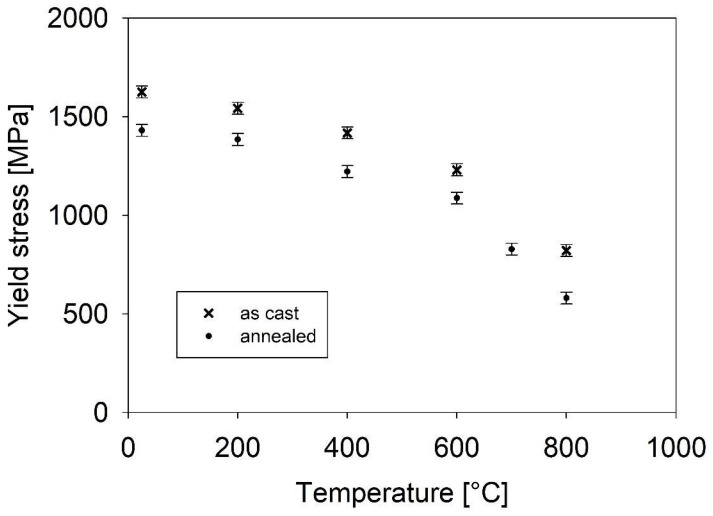
Dependence of the yield stress on the temperature of deformation for the as-cast state and the annealed state.

Liu et al. [42] studied the Peierls–Nabarro stress in the case of CCA. They found that the Peierls–Nabarro stress for CCA is much higher than that of pure metals and approaches the stresses required for plastic deformation. Such a hypothesis was also discussed in [30]. The model of Peierls–Nabbarro stress is realistic and seems to be very promising. The result in Figure 18 supports this model of Peierls–Nabarro stress. A comparison of the dependence of the yield stress on temperature for the as-cast and annealed states, respectively, was made. The yield stress values for the annealed state are about 150–200 MPa lower than for the as-cast state. Annealing of the FeAlCrNiV alloy does not substantially influence the microstructure of both states. In both states, two phases (B2 ordered and bcc phase) with eutectic nanoscale characteristics were observed. As a result, the structure comprises many interfaces between the B2 and bcc phase, leading to the high strength of the material [43]. There are only two differences in the microstructure in the nanoscale range. During annealing, very tiny D03 regions dissolve, and the bcc lattice decomposes into two bcc lattices with very similar lattice parameters (within the margin of error). The absence of very tiny D03 regions causes a decrease in yield stress. Commonly, the driving force for the dissolution of D03 regions and for the decomposition of the bcc lattice into two bcc lattices with very close lattice parameters is related to the reduction in the inner energy and also the inner stresses. Both changes increase the order of atoms in the slip area, and in this way the Peierls–Nabarro stress decreases. Thus, the yield stress should also decrease, which was experimentally observed (Figure 17). Other strengthening features like precipitates, bcc/B2 boundaries, and size of grains, which also play an important role in strengthening, were the same for both states (as-cast and annealed). This explanation just qualitatively discusses the difference in the yield stress for the as-cast and annealed states. The main goal of this research is to study the material microstructure. Because the FeAlNiV alloy seems to be a candidate for engineering applications, a thorough study of the mechanical properties and strengthening mechanisms needs to be performed and is planned in the future. The mechanical properties and thermal stability of the microstructure rank the FeAlCrNiV alloy among materials with high potential for engineering applications at both low and high temperatures.

**Table 3:** Strength and plasticity of alloys from literature. Data were deduced from the deformation curves. The deduction error is 10%.

Alloy	σ0.2 (MPa)/εf (%)	Temperature	σ0.2 (MPa)/εf (%)	Temperature
CoCrFeNiTiAl0 [3]	2460/13	RT	-	-
AlCoCrFeNiV0 [4]	3300/26	RT	-	-
Al12Cr23Fe21Co21Ni23 [8]	370/25	RT	-	-
Al0.5CoCrCuFeNi [11]	1300/5	RT	100/14	800 °C
Al10Co25Cr8Fe15Ni36Ti6 [13]	680/24	RT	310/30	900 °C
Al9V4Cr10Mn12Fe44Ni18 [14]	800/11-17	RT	-	-
Nickel based superalloy [44]	1160/21	RT	1000/21	750 °C
Particle hardening steels [45]	1350/12	RT	-	-

## 4. Conclusions

The FeAlCrNiV complex concentrated alloy with a nearly equiatomic composition was prepared and investigated. The microstructure and mechanical properties of the alloy were characterized by advanced complementary techniques. The following conclusions may be drawn from this study:

- The microstructures of the as-cast and annealed states have a characteristic eutectic mixture of bcc and B2 phase. At the first level, phases separate into matrix and laths approx. 200–300 nm wide and about 1000 nm long. The laths with B2 symmetry are oriented along the [100] direction. The laths are surrounded by a matrix with bcc symmetry. Within one grain, there are several regions with a size of 20–100 µm, in which one region has the structure described above, but in the neighboring region, the crystallographic symmetry of the phases is reversed. This means that the laths have bcc symmetry, while the matrix has B2 symmetry.
- At higher magnification, both phases contain small (20–50 nm) spherical particles of the complementary phase (bcc in B2, B2 in bcc). These seem to have formed only at distances greater than about 100 nm from the phase boundary; therefore, thinner laths or parts of the matrix are free of these spheres. All phases—matrix, laths, and spheres—are crystalographically coherent.
- In the as-cast state, very tiny D03 regions were observed. They are several nanometers in size and, due to this fact, it is very difficult to determine whether these regions have the characteristics of particles, structure domains, etc.
- Annealing at 800 °C for 16 days does not substantially influence the character of the structure. There are two changes connected with the diffusion of atoms: Dissolution of very tiny particles with D03 symmetry and decomposition of the bcc lattice into two lattices with very close lattice parameters (within the margin of error). These nanoscale changes cause a higher order of atoms in the material. However, the substantial features of the structure described above do not change.
- The mechanical properties of the studied EHEA alloy are very good. The yield stress reaches 1625/821 MPa for RT/800 °C for the as-cast state and 1430/580 MPa for RT/800 °C in the annealed state. For both states, the strain to fracture reaches more than 10% for RT and about 28% for 800 °C. Such results nominate this alloy as a very promising candidate for application use.

## Figures and Tables

**Table 1:** Chemical composition determined from the EDX maps in Figure 9 and Figure 13, compared with results of Calphad modeling (using the “TCHEA4” database).

Phase	Alloy	Microstructure	V	Cr	Fe	Al	Ni
			±3 at. %				
**BCC**	annealed	A	29	30	28	7	6
		B	31	33	28	5	3
	as-cast	A	29	31	27	7	5
		B	30	33	27	7	4
	calculated from ThermoCalc 2022a		34	34	25	5	2
**B2**	annealed	A	6	4	9	38	43
		B	8	6	10	38	39
	as-cast	A	9	5	12	36	39
		B	11	8	13	32	36
	calculated from ThermoCalc 2022a		4	1	6	43	46

**Table 2:** Compressive test parameters of the FeAlCrNiV alloy. The symbol “≥” in plasticity data means that the test was stopped due to the technical limits of the deformation machine.

Temperature (°C)	As-Cast			Annealed State		
	σ0.2 (MPa)	σM (MPa)	εf (%)	σ0.2 (MPa)	σM (MPa)	εf (%)
RT	1625 ± 35	2880 ± 55	18 ± 2	1430 ± 30	2380 ± 46	≥12 ± 2
200	1542 ± 30	2450 ± 50	≥15 ± 2	1384 ± 28	2300 ± 45	11 ± 2
400	1417 ± 28	1996 ± 40	≥13 ± 2	1221 ± 24	2000 ± 40	≥13 ± 2
600	1230 ± 25	1677 ± 32	≥15 ± 2	1087 ± 22	1421 ± 30	≥22 ± 2
700	-	-	-	828 ± 20	876 ± 33	≥20 ± 2
800	821 ± 20	680 ± 25	≥24 ± 2	580 ± 15	608 ± 34	≥31 ± 2

## Data Availability

The original contributions presented in this study are included in the article. Further inquiries can be directed to the corresponding author.
